# Apolipoprotein M Inhibits Angiogenic and Inflammatory Response by Sphingosine 1-Phosphate on Retinal Pigment Epithelium Cells

**DOI:** 10.3390/ijms19010112

**Published:** 2017-12-31

**Authors:** Ryo Terao, Megumi Honjo, Makoto Aihara

**Affiliations:** Department of Ophthalmology, Graduate School of Medicine, Tokyo University, Tokyo 113-8654, Japan; qqaf787c9@gmail.com (R.T.); m_honjo@kuhp.kyoto-u.ac.jp (M.H.)

**Keywords:** sphingosine 1-phosphate, sphingolipids and inflammation, S1P-receptors, apolipoprotein M, high-density lipoprotein, choroidal neovascularization, age-related macular degeneration, vascular endothelial growth factor, hypoxia inducible factor, interleukin-8

## Abstract

Sphingosine 1-phosphate (S1P) is a potent lipid mediator that modulates inflammatory responses and proangiogenic factors. It has been suggested that S1P upregulates choroidal neovascularization (CNV) and may be deeply involved in the pathogenesis of exudative age-related macular degeneration (AMD). Recent studies have suggested that apolipoprotein M (ApoM), a carrier protein for S1P, modulates the biological properties of S1P in the pathogenesis of atherosclerosis. However, the role of ApoM/S1P in AMD has not been explored. We investigated the effect of S1P on proangiogenic factors in human retinal pigment epithelium (RPE) cell lines in vitro. S1P promoted the expression of vascular endothelial growth factor in RPE cells. Hypoxia inducible factor-1α expression was also upregulated. These S1P-induced enhancements in growth factors and chemotactic cytokines in RPE cells were significantly inhibited by ApoM treatment. Additionally, in vivo experiments using a laser-induced CNV murine model demonstrated that intravitreal ApoM injection significantly reduced the progression of CNV formation. Although the detailed mechanisms remain to be elucidated, the present results provide a novel potential therapeutic target for AMD, and demonstrate a suppressive role for ApoM and S1P in the pathology of CNV progression.

## 1. Introduction

The retinal pigment epithelium (RPE) is a continuous monolayer of epithelial cells with various functions and is essential for the homeostasis and survival of the overlying photoreceptor cells. It is located between the neurosensory retina and the choroid, and constitutes a single layer of hexanocuboidal pigmented cells [1]. The RPE transports ions, water, and metabolic end products from the subretinal space to the blood [2]. The lateral membrane of RPE cells is integrated by tight junctions that constitute the blood retinal barrier (BRB), and the BRB prevents excess proteins and plasma components from entering the retina. RPE cells secrete many essential factors, including vascular endothelial growth factor (VEGF) [3,4]. RPE cells also play an important role in the immune response by expressing adhesion molecules and cytokines [5]. Therefore, the dysfunction of RPE cells leads to the release of cytokines and retina deterioration, leading to retinal degeneration.

Age-related macular degeneration (AMD) is one of the most common causes of vision loss among people age 50 or older. AMD is characterized by progressive degeneration of the retina, RPE, and the underlying choroid. Choroidal neovascularization (CNV) is responsible for the majority of severe loss of vision in AMD; however, the main cause of the development of CNV is not fully understood [6,7]. In the early stages of the disease, drusen are recognized as yellowish-white spots in the RPE and Bruch’s membrane [8]. Injury to the RPE reportedly causes drusen biogenesis, including age, light damage, oxidative stress, and lipofuscin accumulation, and results in the release of cytokines to Bruch’s membrane. Therefore, neovascular AMD is considered an inflammatory chronic eye disease with damage to RPE cells [9], and it has been reported that several inflammatory cytokines and chemokines are involved in the development of CNV in AMD [10]. In particular, proangiogenic factors, including VEGF, platelet-derived growth factor, fibroblast growth factor, and hypoxia inducible factor (HIF), are also strongly associated with CNV formation [11]. Among these factors, VEGF is considered the most critical promoter of neovascular AMD [12]. The intravitreal injection of anti-VEGF agents has become the first choice for the treatment of neovascular AMD, and is believed to be the most general and effective protection against exacerbation not only in AMD [13,14,15], but also in other sight-threatening diseases accompanied by neovascularization [16,17]. Indeed, anti-VEGF therapy is effective at preventing the progression of CNV, but it shows only a temporal effect, and some AMD patients are resistant to current anti-VEGF therapies [18]. Moreover, because VEGF is essential for cell survival, its excess inhibition leads to chorioretinal atrophy [19], another possible cause of vision loss. Thus, alternative treatment strategies based on more specific targeting of CNV are desirable.

Sphingosine 1-phosphate (S1P) is a vital lysophospholipid mediator. S1P is synthesized via the ATP-dependent phosphorylation of sphingosine by sphingosine kinase [20], and it exerts a wide variety of cellular functions, including cell proliferation, cell survival, cell migration, cellular barrier formation, apoptosis, calcium homeostasis, vascular mutation, and fibrosis, through interactions with its G-protein coupled receptors, S1P1–5 [20,21,22,23]. A previous study showed that S1P was detected in the area of CNV in laser-induced CNV murine models [24]. It has also been reported that intravitreal injections of a humanized monoclonal antibody that specifically binds to S1P reduced the area of CNV and fluorescein leakage from CNV. Therefore, S1P may be important in this chronic inflammatory process and may represent a therapeutic target of AMD. However, there are few reports of the inflammatory and angiogenic effects of S1P in RPE cells.

S1P has been suggested to have pleiotropic effects (i.e., plays both harmful and beneficial roles) in the pathogenesis of atherosclerosis. In the circulation, S1P is carried by albumin (35%) or apolipoprotein M (ApoM) (65%) [25,26,27]. ApoM is one of the component apolipoproteins of high-density lipoprotein (HDL) [28] and reportedly exerts an atheroprotective effect itself [29]. Recent studies have validated that there are different mechanisms between S1P bound to ApoM and S1P bound to albumin [30]. ApoM-bound S1P inhibits endothelial inflammation and restrains monocyte–endothelial adhesion [31]. Another report demonstrated that ApoM-bound S1P exhibited an anti-apoptotic effect on human umbilical vein endothelial cells [32]. However, the biological functions of the ApoM/S1P complex in the pathogenesis of AMD remain unclear. 

In this report, we investigated the effects of S1P on the enhancement of chemokines and proangiogenic factors in RPE cells. Among these factors, interleukin-8 (IL-8), and VEGF were remarkably upregulated in S1P-treated RPE cells and ApoM/S1P significantly reduced their upregulation. These results led us to further investigate the suppressive effects of ApoM/S1P on the development of laser-induced CNV in vivo.

## 2. Results

### 2.1. Expression of Sphingosine 1-Phosphate (S1P) Receptors in Retinal Pigment Epithelium (RPE) Cells

**Figure 1:**
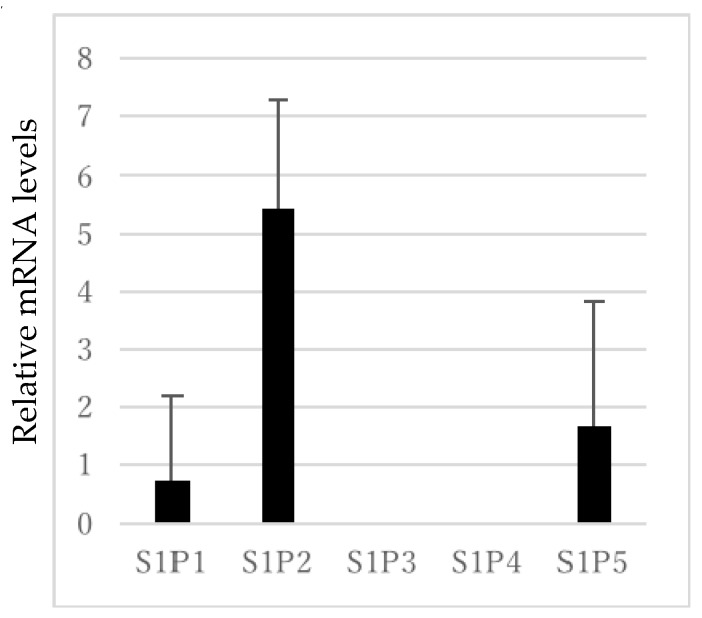
Level of S1P receptors (S1P1–5) mRNA expression normalized to the housekeeping gene glyceraldehyde-3-phosphate dehydrogenase (GAPDH). Each column represents mean SE, *n* = 8.

To determine the effect of S1P in RPE cells, ARPE-19 cells were used in this study. Quantitative real-time polymerase chain reaction (PCR) was performed to detect mRNA expression. First, expression of the S1P receptors (S1P1–5) in ARPE-19 cells was explored. As shown in Figure 1, the expression of S1P1, S1P2, and S1P5 was detected. The expression level of S1P2 was the highest, while S1P4 was not detected and S1P3 was sparely observed.

### 2.2. S1P Upregulates the Expression and Secretion of Vascular Endothelial Growth Factor (VEGF) in RPE Cells

**Figure 2:**
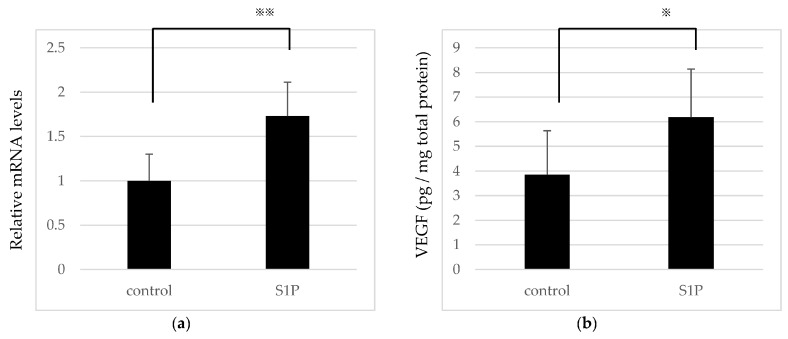
Sphingosine 1-phosphate (S1P) upregulates the expression of vascular endothelial growth factor (VEGF) in ARPE-19 cells. (**a**) Level of VEGF mRNA expression normalized to the housekeeping gene glyceraldehyde-3-phosphate dehydrogenase (GAPDH) (*n* = 5). (**b**) The secretion of VEGF detected by enzyme-linked immunosorbent assay (ELISA) (*n* = 8). Results were expressed as pg/mg total protein. Each column represents mean SE. Statistical analysis was performed using Unpaired Student’s *t*-test. *p* Values were labeled as ^※^ (*p* < 0.05), ^※※^ (*p* < 0.01) vs. control (fatty acid-free bovine serum albumin).

To determine whether S1P induces proangiogenic factors in RPE cells, quantitative real-time PCR and an enzyme-linked immunosorbent assay (ELISA) were used. Several reports have shown that S1P upregulates the expression of VEGF [33]. In this study, ARPE-19 cells were serum-starved for 16 h after reaching confluency and treated with S1P dissolved in fatty acid-free bovine serum albumin (BSA). In preliminary experiments, we have confirmed the concentration-dependent enhancement in VEGF in ARPE-19 cells [34], and Qiao et al. have previous reported the S1P-induced increase of cytokines in ARPE-cells with S1P 5 µM [35], therefore, we stimulated ARPE-19 cells with S1P 5 μM in the present study. The mRNA expression of VEGF was significantly amplified in the presence of 5 μM S1P (Figure 2a). The secretion of VEGF in cell lysates detected by ELISA was also increased (Figure 2b). These results suggest that S1P promotes the pathology of CNV and neovascular AMD by enhancing the release of VEGF by RPE cells. 

### 2.3. S1P Modulates Hypoxia Inducible Factor (HIF)-1α Protein Levels in RPE Cells

**Figure 3:**
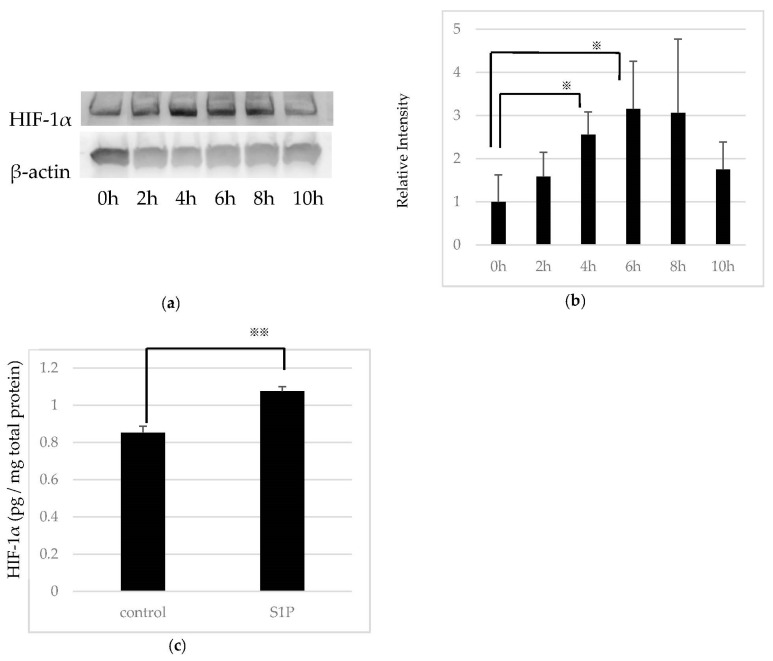
Effect of sphingosine 1-phosphate (S1P) on hypoxia inducible factor (HIF)-1α expression in ARPE-19 cells. (**a**) After starvation, ARPE-19 cells were incubated in the presence of 5 μM S1P. A time-course study showed increased expression of HIF-1α at 4–8 h after treatment. (**b**) Relative band intensity of HIF-1α normalized to β-actin (*n* = 3). (**c**) Detection of the HIF-1α protein level using enzyme-linked immunosorbent assay (ELISA). S1P treatment significantly enhanced HIF-1α accumulation at 6 h after treatment (*n* = 3). Each column represents mean SE. Statistical analysis was performed using Unpaired Student’s *t*-test. *p* Values were labeled as ^※^ (*p* < 0.05), ^※※^ (*p* < 0.01) vs. 0 h or control (fatty acid-free bovine serum albumin).

Since S1P reportedly increases the expression of HIF-1α in vascular endothelial cells [36], we assessed whether exogenous S1P is associated with the regulation of HIF-1α in RPE cells. Treatment of RPE cells with 5-μM S1P increased HIF-1α expression in normoxia (Figure 3a). A time-course study showed that S1P increased HIF-1α levels significantly at 4 h after treatment, which was attenuated after 10 h. The relative band intensity of HIF-1α 4 h and 6 h after the treatment was significantly enhanced compared to that before the treatment (Figure 3b). Likewise, the protein levels of HIF-1α evaluated by ELISA were also significantly increased compared to the control at 6 h after treatment (Figure 3c).

### 2.4. Apolipoprotein M (ApoM)/S1P Inhibits the Expression of Chemokines and Proangiogenic Factors Induced by S1P

**Figure 4:**
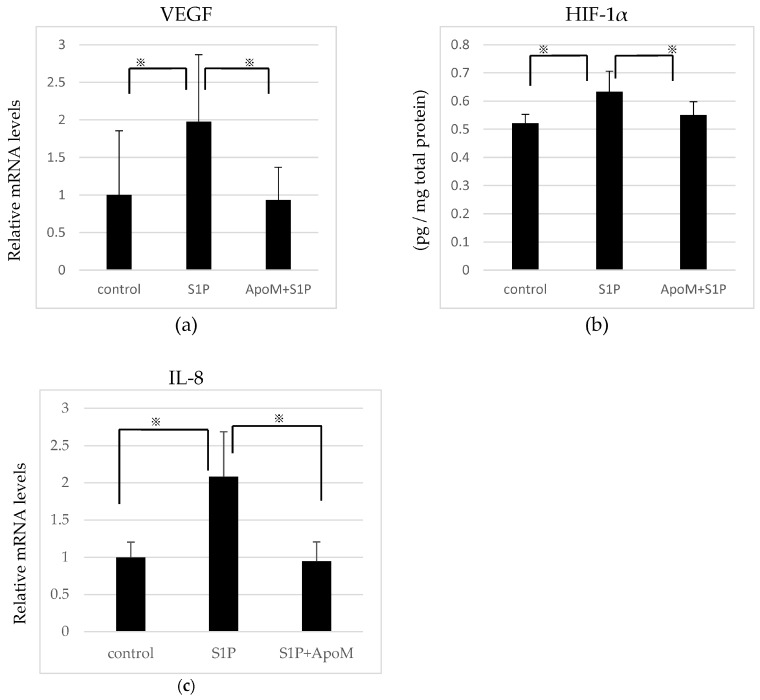
A concentration of 1 μM apolipoprotein M (ApoM) restrained sphingosine 1-phosphate (S1P) (5 μM)-mediated proangiogenic factors and chemokines in ARPE-19 cells. (**a**,**c**) Levels of vascular endothelial growth factor (VEGF) and interleukin (IL)-8 mRNA expression normalized to the housekeeping gene glyceraldehyde-3-phosphate dehydrogenase (GAPDH) (*n* = 12, 6); and (**b**) Hypoxia inducible factor (HIF)-1α protein level detected by enzyme-linked immunosorbent assay (ELISA) (*n* = 7). ApoM significantly attenuated these enhancements induced by treatment with S1P. Each column represents mean SE. Statistical analysis was performed using Tukey test. *p* Values were labeled as ^※^ (*p* < 0.05) vs. control (fatty acid-free bovine serum albumin).

We next analyzed the effect of ApoM-bound S1P in RPE cells. We compared the expression of proangiogenic factors, which was upregulated by albumin-bound S1P stimulation as described above, in the presence of 1 μM ApoM with albumin-bound S1P. ApoM is a member of the Lipocalin family, which has a calyx-like pocket binding to S1P [37]. ApoM is secreted by hepatocytes and stored in the blood plasma. The plasma concentration of ApoM is approximately 0.9 μM [38]. In this present study, we used recombinant ApoM at a concentration comparable to the plasma. Treatment with ApoM only did not show any effects on the expression of proangiogenic factors [34]. A concentration of 1 μM ApoM significantly mitigated the mRNA expression of VEGF promoted by 5 μM S1P (Figure 4a). ApoM also significantly attenuated expression of the HIF-1α protein (Figure 4b). A previous study documented that RPE cells expressed and secreted IL-8 under the influence of S1P [35]. We examined the effect of ApoM/S1P on the expression of IL-8 in RPE cells and observed that ApoM also significantly inhibited the enhancement of IL-8 mRNA stimulated by albumin-bound S1P (Figure 4c).

### 2.5. ApoM/S1P Suppresses the Enhancement of Laser-Induced Choroidal Neovascularization (CNV) in Mice

**Figure 5:**
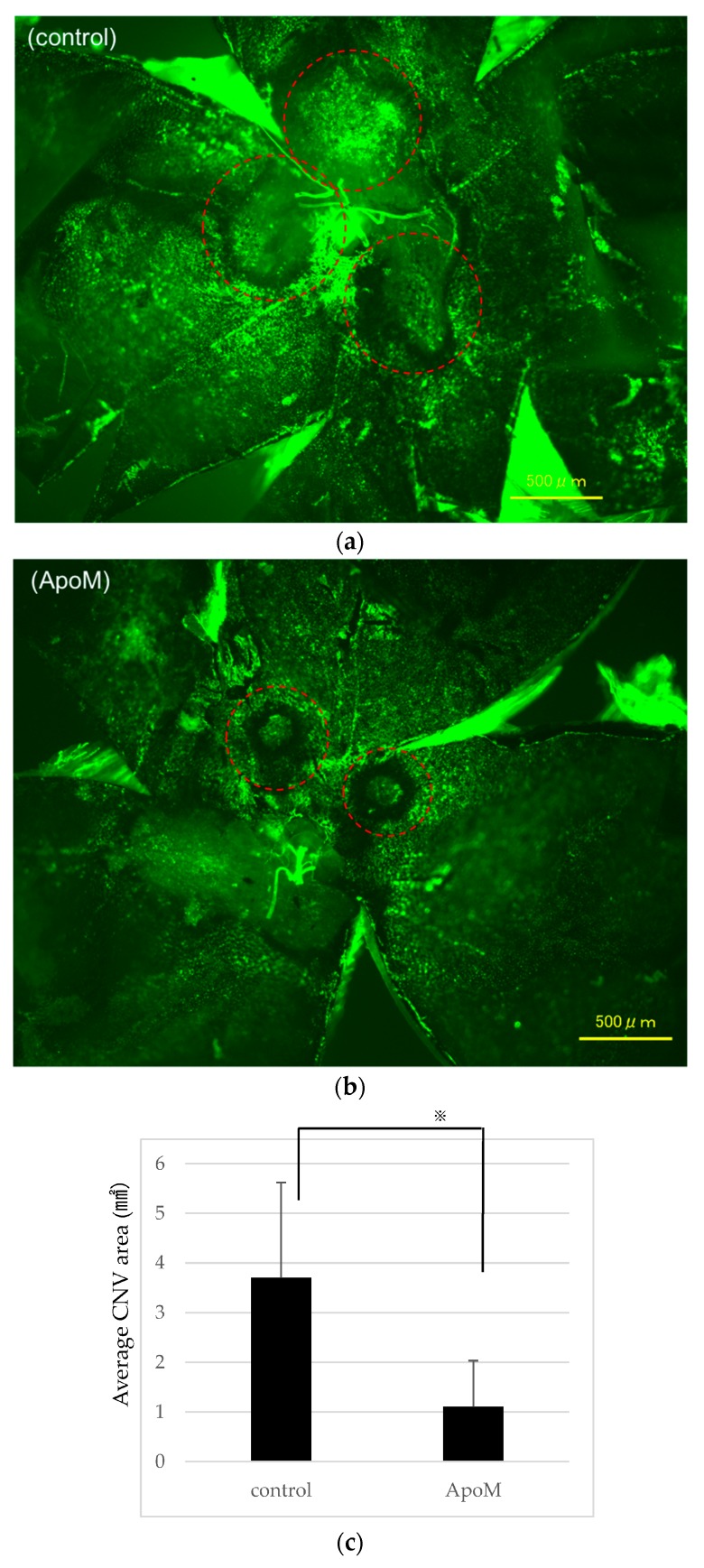
Intravitreal ApoM injection inhibited choroidal neovascularization (CNV) growth in a murine model. (**a**,**b**) CNV was visualized in flat-mounted choroid by fluorescein in laser-induced CNV model mice treated with: vehicle (**a**); or 1 μM recombinant apolipoprotein M (ApoM) (**b**). The red ellipses outline the area of CNV. (**c**) CNV lesion volumes were significantly decreased in ApoM-treated mice compared with control mice. Each column represents mean SE, *n* = 8. Statistical analysis was performed using Unpaired Student’s *t*-test. *p* Values were labeled as ^※^ (*p* < 0.05) vs. control (phosphate-buffered saline ).

Based on the in vitro results, we investigated whether ApoM is responsible for the inhibitory effect of S1P in vivo. We examined whether ApoM had an antiangiogenic effect using a laser-induced CNV model. Intravitreal injection of 1 μM ApoM markedly suppressed the progression of CNV formation (Figure 5a,b). According to the statistical analysis, intravitreal administration of ApoM significantly suppressed CNV progression compared to the control (Figure 5c). 

## 3. Discussion

In the present study, we found that S1P mediates the proangiogenic reaction in RPE cells. Following S1P treatment, the expression levels of HIF-1α and VEGF, both of which are considered to be strongly associated with the development of neovascular AMD, increased. Moreover, ApoM suppressed the expression of these growth factors and chemotactic cytokines induced by S1P. Thus, ApoM/S1P could exert an inhibitory effect on the pathogenesis of CNV. 

To date, previous reports have demonstrated that S1P is a notable lipid mediator involved in various responses and has been accepted as an angiogenic factor [39,40]. However, only a few studies have investigated the biological role of S1P in RPE cells, especially on exudative AMD. S1P stimulates the expression of IL-8 in ARPE-19 cells [35], and induces the proliferation and fibrosis of RPE cells [41]. Our present data also clearly demonstrated the proangiogenic response induced by S1P in RPE cells, as S1P may induce angiogenesis associated with exudative AMD by upregulating VEGF levels, as well as HIF-1α, in RPE cells. The current results, together with those from previous studies, support a role for S1P in retinal CNV and possibly in the pathogenesis of AMD. Thus, an important issue for future clinical research will be to address whether S1P is upregulated in AMD patients. 

A previous study used ARPE-19 cells for stimulation with S1P 5 µM [35], and found the dose-dependent enhancement in the expression of IL-8. In addition, we confirm concentration-dependent enhancement in VEGF in the introductory investigation [34]. Obtaining these results and according to the previous reports, we used 5 µM S1P. S1P is released from the vascular endothelium and blood cells, especially erythrocytes, thrombocytes, and mast cells [26], and S1P concentrations in the plasma range up to 1 μM [42]. The exact source of S1P in ocular tissues remains unknown, but it may originate from ocular blood vessels, possibly from retinal or choroidal vessels. Skoura et al. reported only low levels (5–30 pmol/mg of protein) of S1P in retinal tissues of a murine ischemia model [43]. Although hypoxia or ischemia has been implicated as an important cue for the development of neovascularization, the permeability changes of retinal and choroidal vessels and the inflammatory responses are also greatly involved in AMD. Therefore, further studies will be needed to evaluate the retinal level of S1P and its involvement in AMD pathogenesis. 

S1P upregulates chemotactic cytokines and VEGF in vascular endothelial cells and mast cells [33]. According to previous studies, there are close interactions between S1P signaling and that mediated by VEGF. S1P-induced angiogenesis is mediated by VEGF receptors [44,45]. In addition, VEGF upregulates the expression of S1P1 receptors and S1P-mediated downstream signaling [46], and causes the translocation and activation of sphingosine kinase, resulting in increased S1P generation [47,48].

As shown in Figure 1, the S1P1, S1P2, and S1P5 receptors are expressed in ARPE-19 cells. Among them, the expression level of S1P2 was the highest, corroborating a previous report in which the S1P2 receptor was induced during ischemia-driven retinopathy in mice, peaking at the growth phase of pathologic neovascularization, in which VEGF also plays an important role [43]. It is believed that S1P2 facilitates abnormal retinal angiogenesis and vascular permeability, and that inhibitors of S1P2 may be a therapeutic target for pathologic neovascularization. Our result of the distribution of S1P receptors in ARPE-19 cells was slightly different from that of a previous report [35], in which the expression of S1P3 was the highest in all S1P receptors. However, our result of the S1PR expression was more analogous pattern to the human primary RPE cells described in the report by Swaney et al. In this report, S1P1, -2, -3 and -5 were expressed and the expression of S1P2 seems to be the highest, which is in good accordance with our results. There may exist individual differences in expression pattern of S1P receptors, and further studies should be explored. 

Chumanevich et al. reported that S1P activated VEGF and matrix metalloproteinase-2 in human primary mast cells, which were inhibited by the selective S1P2 antagonist JTE-013 [49]. Sanchez et al. demonstrated that S1P2 increased vascular permeability and disrupted adherens junctions and cellular barrier function using S1P2-transduced human umbilical vein endothelial cells [50]. Cellular junction disruption is partly caused by the Tyr phosphorylation of VE-cadherin, which is also induced by VEGF. In this report, JTE-013 inhibited barrier dysfunction induced by S1P2. They also found that activated S1P2 induced the Rho/Rock/phosphatase and tensin homolog deleted on chromosome 10 pathways. In vivo, knockout of the S1P2 receptor has been shown to mitigate cellular inflammation, vascular growth, and permeability in a murine model of oxygen-induced retinopathy [43]. As in previous reports, we demonstrated that S1P enhanced the expression of VEGF, leading to the development of CNV. These proangiogenic reactions may be attributable to the effects observed downstream of S1P2.

According to Qiao et al., the secretion of IL-8 enhanced by S1P is mediated by p38 mitogen-activated protein kinase (MAPK) pathways and the extracellular-regulated protein kinases 1/2 (ERK1/2) [35]. Other reports have reported that S1P-induced IL-8 secretion is regulated by S1P2 and nuclear factor kappa B in human bronchial epithelial cell lines [51]. It is hypothesized that S1P is correlated with chronic inflammatory pathogenesis in RPE cells, likely via S1P/S1P2 signaling.

S1P also regulates HIF-1α expression [36]. In normoxia, HIF-α prolyl hydroxylases hydroxylate one or two proline residues in the oxygen-dependent degradation domain of HIF-α. Hydroxylated HIF-α binds to the von Hippel–Lindau tumor suppressor protein; this complex undergoes poly-ubiquitination, followed by proteasomal degradation [52]. Conversely, in hypoxia, PHD activity is attenuated. HIF-α is stabilized and translocated to the nucleus. Consequently, the HIF-1 complex binds to the hypoxia response element sequence, inducing erythropoietin, phosphoglycerate kinase-1, and VEGF. Previous study has already reported the hypoxia-induced expression of HIF-1α and VEGF in RPE cells [53,54]. It has been also demonstrated there are several nonhypoxic regulators that affect either the accumulation or activity of HIF-1, including reactive oxygen species, IL-1β, tumor necrosis factor-α (TNF-α), and lipopolysaccharides. The mechanism of HIF-1α accumulation by S1P stimulation has not yet been elucidated. It is believed that S1P increases HIF-1α protein expression via phosphoinositide 3-kinase, a mammalian target of rapamycin, MAP/ERK kinase, and protein kinase C βI [55]. In our study, we determined that S1P increases HIF-1α accumulation in RPE cells (Figure 3). These results suggest that S1P is one of the non-hypoxic activators of HIF-1α in RPE cells, and regulates HIF-1-dependent cellular responses. Michaud et al. reported that S1P stabilized HIF-1α protein in a time- and dose-dependent manner via a pVHL-independent pathway in vascular endothelial cells [36]. The current report also demonstrated that S1P2 regulates HIF-1α expression. Therefore, we believe that S1P/S1P2 also play a crucial role in RPE cells. 

Our research showed that S1P enhanced the bioactive factors described above (IL-8, HIF-1α, and VEGF) that play important roles in the pathogenesis of neovascularization [56]. We established S1P as a pivotal mediator that associates with CNV lesions. Furthermore, together with the reports mentioned above, we presumed that S1P, especially S1P/S1P2 signaling, plays an important role in inflammation and the angiogenic process in CNV progression. Therefore, we next investigated how ApoM behaves toward S1P and S1P receptors as a primary carrier of S1P on RPE cells and in a murine laser-induced CNV model, and explored the effect of ApoM/S1P on CNV progression in vivo. 

Several reports have suggested different mechanisms between ApoM-bound S1P and albumin-bound S1P [37]. Ruiz et al. demonstrated that ApoM-bound S1P restrained the TNF-α-induced expression of adhesion molecules (E-selectin, intercellular adhesion molecule-1, and vascular adhesion molecule-1) in primary human aortic endothelial cells (HAECs) [31]. In the same report, they revealed that ApoM-bound S1P attenuated monocyte–endothelial adhesion in HAECs. Similar results were shown using purified HDL from human plasma, where the majority of ApoM exists. The authors indicated that ApoM-bound S1P reduces endothelial inflammation via S1P1 signaling, which promotes vascular barrier function and the anti-inflammatory response. Another report also found that ApoM improves the endothelial barrier function via S1P/S1P1 signaling [57]. In vivo, HDL-bound S1P mitigates lymphopoiesis and neuroinflammation in mice [58]. Moreover, ApoM^−/−^ mice exhibit increased vascular endothelial permeability in the lung [59]. Hence, based on previous reports, we hypothesize that the suppressive effect of ApoM on inflammation is derived from the activation of S1P/S1P1 or from the inhibition of S1P/S1P2. In addition, ApoM may exert a crucial role as an inhibitor of CNV by suppressing inflammation and the proangiogenic response by RPE cells. In fact, our study showed that the administration of recombinant ApoM restrained the S1P-induced activation of growth factors and chemokines (Figure 4). Although the detailed mechanisms remain to be elucidated, this hypothesis was confirmed, at least in part, both in vivo and in vitro.

There are some limitations to this study. First, we used ARPE-19 cells instead of human RPE cells. An immortalized cell line may exert a different response against S1P and ApoM-bound S1P. Second, the presence of S1P and ApoM in the retina must be clarified in vivo using an animal model or AMD patients. The quantification of a small amount of lipid will be a challenge. In the future, we plan to elucidate the S1P, ApoM, and S1P receptor distributions in AMD patients. Third, the mechanism of ApoM/S1P as a ligand of S1P1 or as an inhibitor of S1P2 remains unknown. Further investigation is needed to clarify the role of S1P-dependent angiogenesis in the retina. Fourth, we did not compare the concentration of exogenous and endogenous S1P. Intracellular S1P level in RPE cells has not yet been determined but we are currently conducting the study for verification, which should be investigated in future studies. Finally, we showed data on a single member of the chemotactic cytokine family (IL-8) on the suppressive effect of ApoM/S1P. Additional data on other cytokines need to be acquired from future studies. We will continue this work in future study.

Even though more experiments on the physiological pathway involving each S1P receptor in RPE cells are required, the present study indicates that ApoM may be used as an alternative treatment strategy for CNV by targeting S1P.

## 4. Materials and Methods 

### 4.1. Culture of RPE Cells

The human RPE cell line ARPE-19 (American Type Culture Collection, Manassas, VA, USA) was used for the experiments described herein. Cells were cultured in Dulbecco’s Modified Eagle’s Medium/F12 (DMEM/F12) (Sigma-Aldrich, St. Louis, MO, USA) supplemented with 10% fetal bovine serum, 1% penicillin, and 1% streptomycin. Cultures were incubated at 37 °C in a humidified atmosphere of 5% CO_2_. Medium was replaced every 2 days. Cells between passages 3 and 26 were used in our experiments. Upon reaching confluency, cells were serum-starved for 16–20 h before experiments. 

### 4.2. Reagents

S1P was purchased from Enzo Life Sciences, Inc. (Exeter, UK). Recombinant human and murine ApoM was purchased from R&D Systems (Minneapolis, MN, USA). Anti-HIF-1α antibody was purchased from Invitrogen (Carlsbad, CA, USA). Anti-β-actin antibody was purchased from Gene Tex (San Antonio, TX, USA). Primers for real-time PCR were purchased from Hokkaido System Science Co., Ltd. (Sapporo, Japan).

### 4.3. Preparation of S1P and ApoM-Bound S1P

S1P in methanol (0.5 mg/mL) was stored at −20 °C. When used in the experiments, the methanol was evaporated with nitrogen gas. The remaining S1P deposit was dissolved in bovine serum albumin (fatty free, 4 mg/mL). 

The ApoM/S1P complex was loaded as previously described [31]. The necessary quantity of S1P was dissolved in 1 μM human recombinant ApoM and incubated for 45 min at room temperature. No desalting procedure was performed during this process. 

### 4.4. Quantitative Real-Time Polymerase Chain Reaction (PCR)

RNA was isolated from treated cells using TRIzol reagent (Molecular Research Center, Inc., Cincinnati, OH, USA) according to the manufacturer’s instructions. Total RNA concentration was measured by reading the absorbance at 260 nm. Each sample was reverse-transcribed into cDNA with ReverTra Ace^®^ qPCR RT Master Mix with gDNA Remover (TOYOBO Co., Ltd., Life Science Department, Osaka, Japan). Real-time PCR was performed with SYBR Premix Ex Taq II (Tli RNaseH Plus; TaKaRa BIO, Inc., Shiga, Japan) and the Thermal Cycler Dice Real Time System II (TaKaRa BIO, Inc.) according to the manufacturer’s instructions. Relative mRNA expression values of the genes of interest were normalized to the housekeeping gene glyceraldehyde-3-phosphate dehydrogenase (GAPDH). PCR primers used to amplify S1P1-5, IL-8, VEGF, and GAPDH are summarized in Table 1. 

### 4.5. Western Blotting

After treatment, cultured ARPE-19 cells were rinsed twice with phosphate-buffered saline (PBS). Protein lysates were isolated from treated cells using ice-cold radioimmunoprecipitation buffer (Thermo Fisher Scientific K.K., Yokohama, Japan) containing protease inhibitor cocktail tablets (Roche Diagnostics, Basel, Switzerland). The samples were collected in CryoTubes (Sumitomo Bakelite Co., Ltd., Tokyo, Japan), sonicated, and centrifuged for 20 min at 15,000 rpm at 4 °C. The supernatants were then used for subsequent analyses. Total protein amounts were calculated using the Pierce BCA Protein Assay (Thermo Fisher Scientific K.K.). After the addition of loading buffer and 10% β-mercaptoethanol, the samples were denatured by heating at 75 °C for 10 min. Next, an equal amount of cellular protein was electrophoresed with sodium dodecyl sulfate polyacrylamide gel and transferred to polyvinylidene difluoride membranes (BIO-RAD Laboratories, Hercules, CA, USA). After washing with Tris-buffered saline containing Tween-20 (TBST) for 5 min, blots were incubated in blocking solution (Nacalai Tesque, Inc., Kyoto, Japan) for 30 min at room temperature. The membranes were incubated with primary antibodies overnight at 4 °C. On the following day, membranes were washed thrice with TBST for 5 min, and incubated with horseradish peroxidase-conjugated anti-mouse or anti-rabbit secondary antibodies (1:2000–1:5000; Thermo Fisher Scientific K.K.) for 1 h at room temperature. Protein bands were detected using an ImageQuant LAS 4000 mini (GE Healthcare, Chicago, IL, USA). Bands were quantified using ImageJ 1.49 (National Institutes of Health, Rockville, MD, USA). Anti-HIF-1α (1:500) and β-actin (1:2000) were used as primary antibodies. β-actin was used as a loading control. The Western blotting band intensities were measured with Multi Gauge ver. 3.1 software (FUJIFILM Co., Tokyo, Japan).

### 4.6. Enzyme-Linked Immunosorbent Assay (ELISA)

To evaluate the expression of VEGF and HIF-1α in cell lysates, the Quantikine^®^ Human Immunoassay Kit (R&D Systems) was used according to the manufacturer’s instructions. Each concentration was measured by the color intensity of solution using a microplate reader (2030 ARVO X3, Perkin Elmer Japan, Kanagawa, Japan). Concentrations were calculated by comparing the standard curve using known concentrations. Results are expressed as pg/mg protein.

### 4.7. Murine Model of CNV

All animal experiments were performed in accordance with the ethical guidelines for animal experimentation of the University of Tokyo and the Association for Research in Vision and Ophthalmology statement for the Use of Animals in Ophthalmologic and Vision Research.

Eight-week-old pathogen-free male C57BL/6J mice were purchased from Tokyo Laboratory Animals Science Co., Ltd. (Tokyo, Japan). Mice were deeply anesthetized by intraperitoneal injection of a mixture of ketamine (80 mg/kg; Daiichi Sankyo Propharma Co., Ltd., Tokyo, Japan) and xylazine (16 mg/kg; Bayer, Leverkusen, Germany) in sterile saline. Pupils were dilated by topical administration of a 0.5% tropicamide-phenylephrine hydrochloride combination (Santen, Osaka, Japan).

Laser-induced CNV was generated following a documented protocol [60]. CNV lesions in both eyes were generated with a diode laser (DC-3300, Nidec Co., Ltd., Aichi, Japan) coupled to a slit lamp set for a 75 μm spot, 150 mW intensity, and 50 ms duration. When Bruch’s membrane ruptured, a characteristic vapor bubble formed [60]. Lesions that induced apparent hemorrhage were excluded from this study. Immediately after laser induction, intravitreal injection was performed. One eye was treated with recombinant murine ApoM (1 μg/μm, 1 μL), and the other eye was treated with an equal dose of vehicle (PBS) as a control. 

Mice were sacrificed 7 days after treatment. Mice were euthanized by an overdose of the drugs mentioned above. Animals then underwent whole-body perfusion via cardiac puncture with fluorescein-conjugated concanavalin A (Vector Laboratories, Inc., Burlingame, CA, USA). The eyes were enucleated and dissected to isolate the posterior segment containing the RPE, choriocapillaris, and sclera. The area of CNV lesions was measured.

### 4.8. Statistical Analysis

Differences between two groups were compared using the Student’s *t*-test. Differences between three groups were analyzed by the Tukey test. *p* ≤ 0.05 indicated statistical significance. Data and figures are depicted as the mean ± standard error of the mean.

## 5. Conclusions

In the present study, we demonstrated that S1P is a potent lipid mediator that modulates proangiogenic factors in RPE cells. ApoM effectively inhibits the expression of proangiogenic factors and chemokines and suppresses the progression of CNV formation. These findings may be helpful in the development of a new treatment for AMD.

## Figures and Tables

**Table 1:** DNA primers used for real-time PCR.

Oligos	Forward (5′–3′)	Reverse (3′–5′)
S1P1	AAATTCCACCGACCCATGTA	AGTTATTGCTCCCGTTGTGG
S1P2	ACTGTCCTGCCTCTCTACGCC	GTCTTGAGCAGGGCTAGCGTC
S1P3	ACCATCGTGATCCTCTACGCAC	CTTGATTTACTTCTGCTTGGGTCG
S1P4	TGCTGAAGACGGTGCTGATG	CCCAGAGGTTGGAGCCAAAG
S1P5	AGGACCTTGTGGGTGATATAGAGGAC	CCCCTTCACCTTCTCTGGTTTTC
IL-8	ATGACTTCCAAGCTGGCCGTGGCT	TCTCAGCCCTCTTCAAAAACTTCTC
VEGF	CCCTGATGAGATCGAGTACATCT	AGCAAGGCCCACAGGGATTT
GAPDH	AATTCCATGGCACCGTCAAG	ATCGCCCCACTTGATTTTGG

## References

[1] Sparrow J.R., Hicks D., Hamel C.P. (2010). The retinal pigment epithelium in health and disease. Curr. Mol. Med..

[2] Dornonville de la Cour M. (1993). Ion transport in the retinal pigment epithelium. A study with double barrelled ion-selective microelectrodes. Acta Ophthalmol. Suppl..

[3] Liljekvist-Soltic I., Olofsson J., Johansson K. (2008). Progenitor cell-derived factors enhance photoreceptor survival in rat retinal explants. Brain Res..

[4] Blaauwgeers H.G., Holtkamp G.M., Rutten H., Witmer A.N., Koolwijk P., Partanen T.A., Alitalo K., Kroon M.E., Kijlstra A., van Hinsbergh V.W. (1999). Polarized vascular endothelial growth factor secretion by human retinal pigment epithelium and localization of vascular endothelial growth factor receptors on the inner choriocapillaris. Evidence for a trophic paracrine relation. Am. J. Pathol..

[5] Holtkamp G.M., Kijlstra A., Peek R., de Vos A.F. (2001). Retinal pigment epithelium-immune system interactions: Cytokine production and cytokine-induced changes. Prog. Retin. Eye Res..

[6] Ferris F.L., Fine S.L., Hyman L. (1984). Age-related macular degeneration and blindness due to neovascular maculopathy. Arch. Ophthalmol..

[7] Klein R., Wang Q., Klein B.E., Moss S.E., Meuer S.M. (1995). The relationship of age-related maculopathy, cataract, and glaucoma to visual acuity. Investig. Ophthalmol. Vis. Sci..

[8] Sarks S.H. (1976). Ageing and degeneration in the macular region: A clinico-pathological study. Br. J. Ophthalmol..

[9] Nowak J.Z. (2006). Age-related macular degeneration (AMD): Pathogenesis and therapy. Pharmacol. Rep..

[10] Nagineni C.N., Kommineni V.K., Ganjbaksh N., Nagineni K.K., Hooks J.J., Detrick B. (2015). Inflammatory cytokines induce expression of chemokines by human retinal cells: Role in chemokine receptor mediated age-related macular degeneration. Aging Dis..

[11] Cabral T., Mello L.G.M., Lima L.H., Polido J., Regatieri C.V., Belfort R., Mahajan V.B. (2017). Retinal and choroidal angiogenesis: A review of new targets. Int. J. Retin. Vitreous.

[12] Bressler S.B. (2009). Introduction: Understanding the role of angiogenesis and antiangiogenic agents in age-related macular degeneration. Ophthalmology.

[13] Heier J.S., Brown D.M., Chong V., Korobelnik J.F., Kaiser P.K., Nguyen Q.D., Kirchhof B., Ho A., Ogura Y., Yancopoulos G.D. (2012). Intravitreal aflibercept (VEGF trap-eye) in wet age-related macular degeneration. Ophthalmology.

[14] Rosenfeld P.J., Brown D.M., Heier J.S., Boyer D.S., Kaiser P.K., Chung C.Y., Kim R.Y. (2006). Ranibizumab for neovascular age-related macular degeneration. N. Engl. J. Med..

[15] Schmidt-Erfurth U., Chong V., Loewenstein A., Larsen M., Souied E., Schlingemann R., Eldem B., Mones J., Richard G., Bandello F. (2014). Guidelines for the management of neovascular age-related macular degeneration by the European society of retina specialists (EURETINA). Br. J. Ophthalmol..

[16] Mintz-Hittner H.A., Kennedy K.A., Chuang A.Z. (2011). Efficacy of intravitreal bevacizumab for stage 3+ retinopathy of prematurity. N. Engl. J. Med..

[17] Massin P., Bandello F., Garweg J.G., Hansen L.L., Harding S.P., Larsen M., Mitchell P., Sharp D., Wolf-Schnurrbusch U.E., Gekkieva M. (2010). Safety and efficacy of ranibizumab in diabetic macular edema (RESOLVE study): A 12-month, randomized, controlled, double-masked, multicenter phase II study. Diabetes Care.

[18] Stangos A.N., Gandhi J.S., Nair-Sahni J., Heimann H., Pournaras C.J., Harding S.P. (2010). Polypoidal choroidal vasculopathy masquerading as neovascular age-related macular degeneration refractory to ranibizumab. Am. J. Ophthalmol..

[19] Fernandez-Robredo P., Sancho A., Johnen S., Recalde S., Gama N., Thumann G., Groll J., Garcia-Layana A. (2014). Current treatment limitations in age-related macular degeneration and future approaches based on cell therapy and tissue engineering. J. Ophthalmol..

[20] Spiegel S., Milstien S. (2003). Sphingosine-1-phosphate: An enigmatic signalling lipid. Nat. Rev. Mol. Cell Biol..

[21] Sanchez T., Hla T. (2004). Structural and functional characteristics of S1P receptors. J. Cell. Biochem..

[22] Lee J.F., Zeng Q., Ozaki H., Wang L., Hand A.R., Hla T., Wang E., Lee M.J. (2006). Dual roles of tight junction-associated protein, zonula occludens-1, in sphingosine 1-phosphate-mediated endothelial chemotaxis and barrier integrity. J. Biol. Chem..

[23] Takabe K., Paugh S.W., Milstien S., Spiegel S. (2008). “Inside-out” Signaling of sphingosine-1-phosphate: Therapeutic targets. Pharmacol. Rev..

[24] Xie B., Shen J., Dong A., Rashid A., Stoller G., Campochiaro P.A. (2009). Blockade of sphingosine-1-phosphate reduces macrophage influx and retinal and choroidal neovascularization. J. Cell. Physiol..

[25] Aoki S., Yatomi Y., Ohta M., Osada M., Kazama F., Satoh K., Nakahara K., Ozaki Y. (2005). Sphingosine 1-phosphate-related metabolism in the blood vessel. J. Biochem..

[26] Murata N., Sato K., Kon J., Tomura H., Yanagita M., Kuwabara A., Ui M., Okajima F. (2000). Interaction of sphingosine 1-phosphate with plasma components, including lipoproteins, regulates the lipid receptor-mediated actions. Biochem. J..

[27] Christoffersen C., Obinata H., Kumaraswamy S.B., Galvani S., Ahnstrom J., Sevvana M., Egerer-Sieber C., Muller Y.A., Hla T., Nielsen L.B. (2011). Endothelium-protective sphingosine-1-phosphate provided by HDL-associated apolipoprotein M. Proc. Natl. Acad. Sci. USA.

[28] Xu N., Dahlback B. (1999). A novel human apolipoprotein (APOM). J. Biol. Chem..

[29] Christoffersen C., Jauhiainen M., Moser M., Porse B., Ehnholm C., Boesl M., Dahlback B., Nielsen L.B. (2008). Effect of apolipoprotein m on high density lipoprotein metabolism and atherosclerosis in low density lipoprotein receptor knock-out mice. J. Biol. Chem..

[30] Kurano M., Tsukamoto K., Ohkawa R., Hara M., Iino J., Kageyama Y., Ikeda H., Yatomi Y. (2013). Liver involvement in sphingosine 1-phosphate dynamism revealed by adenoviral hepatic overexpression of apolipoprotein M. Atherosclerosis.

[31] Ruiz M., Frej C., Holmer A., Guo L.J., Tran S., Dahlback B. (2017). High-density lipoprotein-associated apolipoprotein m limits endothelial inflammation by delivering sphingosine-1-phosphate to the sphingosine-1-phosphate receptor 1. Arterioscler. Thromb. Vasc. Biol..

[32] Ruiz M., Okada H., Dahlback B. (2017). HDL-associated ApoM is anti-apoptotic by delivering sphingosine 1-phosphate to S1P1 & S1P3 receptors on vascular endothelium. Lipids Health Dis.

[33] Hait N.C., Oskeritzian C.A., Paugh S.W., Milstien S., Spiegel S. (2006). Sphingosine kinases, sphingosine 1-phosphate, apoptosis and diseases. Biochim. Biophys. Acta.

[34] Terao R. (2017).

[35] Qiao Y., Hu R., Wang Q., Qi J., Yang Y., Kijlstra A., Yang P. (2012). Sphingosine 1-phosphate elicits proinflammatory responses in ARPE-19 cells. Investig. Ophthalmol. Vis. Sci..

[36] Michaud M.D., Robitaille G.A., Gratton J.P., Richard D.E. (2009). Sphingosine-1-phosphate: A novel nonhypoxic activator of hypoxia-inducible factor-1 in vascular cells. Arterioscler. Thromb. Vasc. Biol..

[37] Hajny S., Christoffersen C. (2017). A novel perspective on the ApoM-S1P axis, highlighting the metabolism of ApoM and its role in liver fibrosis and neuroinflammation. Int. J. Mol. Sci..

[38] Axler O., Ahnstrom J., Dahlbock B. (2007). An elisa for apolipoprotein m reveals a strong correlation to total cholesterol in human plasma. J. Lipid Res..

[39] Hla T. (2004). Physiological and pathological actions of sphingosine 1-phosphate. Semin. Cell Dev. Biol..

[40] Maines L.W., French K.J., Wolpert E.B., Antonetti D.A., Smith C.D. (2006). Pharmacologic manipulation of sphingosine kinase in retinal endothelial cells: Implications for angiogenic ocular diseases. Investig. Ophthalmol. Vis. Sci..

[41] Swaney J.S., Moreno K.M., Gentile A.M., Sabbadini R.A., Stoller G.L. (2008). Sphingosine-1-phosphate (S1P) is a novel fibrotic mediator in the eye. Exp. Eye Res..

[42] Egom E.E., Fitzgerald R., Canning R., Pharithi R.B., Murphy C., Maher V. (2017). Determination of sphingosine-1-phosphate in human plasma using liquid chromatography coupled with Q-ToF mass spectrometry. Int. J. Mol. Sci..

[43] Skoura A., Sanchez T., Claffey K., Mandala S.M., Proia R.L., Hla T. (2007). Essential role of sphingosine 1-phosphate receptor 2 in pathological angiogenesis of the mouse retina. J. Clin. Investig..

[44] Endo A., Nagashima K., Kurose H., Mochizuki S., Matsuda M., Mochizuki N. (2002). Sphingosine 1-phosphate induces membrane ruffling and increases motility of human umbilical vein endothelial cells via vascular endothelial growth factor receptor and CrkII. J. Biol. Chem..

[45] Tanimoto T., Jin Z.G., Berk B.C. (2002). Transactivation of vascular endothelial growth factor (VEGF) receptor Flk-1/KDR is involved in sphingosine 1-phosphate-stimulated phosphorylation of Akt and endothelial nitric-oxide synthase (eNOS). J. Biol. Chem..

[46] Igarashi J., Erwin P.A., Dantas A.P., Chen H., Michel T. (2003). VEGF induces S1P1 receptors in endothelial cells: Implications for cross-talk between sphingolipid and growth factor receptors. Proc. Natl. Acad. Sci. USA.

[47] Pitson S.M., Xia P., Leclercq T.M., Moretti P.A., Zebol J.R., Lynn H.E., Wattenberg B.W., Vadas M.A. (2005). Phosphorylation-dependent translocation of sphingosine kinase to the plasma membrane drives its oncogenic signalling. J. Exp. Med..

[48] Hernandez-Coronado C.G., Guzman A., Rodriguez A., Mondragon J.A., Romano M.C., Gutierrez C.G., Rosales-Torres A.M. (2016). Sphingosine-1-phosphate, regulated by FSH and VEGF, stimulates granulosa cell proliferation. Gen. Comp. Endocrinol..

[49] Chumanevich A., Wedman P., Oskeritzian C.A. (2016). Sphingosine-1-phosphate/sphingosine-1-phosphate receptor 2 axis can promote mouse and human primary mast cell angiogenic potential through upregulation of vascular endothelial growth factor-a and matrix metalloproteinase-2. Mediat. Inflamm..

[50] Sanchez T., Skoura A., Wu M.T., Casserly B., Harrington E.O., Hla T. (2007). Induction of vascular permeability by the sphingosine-1-phosphate receptor-2 (S1P2R) and its downstream effectors ROCK and PTEN. Arterioscler. Thromb. Vasc. Biol..

[51] O’Sullivan M.J., Hirota N., Martin J.G. (2014). Sphingosine 1-phosphate (S1P) induced interleukin-8 (IL-8) release is mediated by S1P receptor 2 and nuclear factor κb in beas-2b cells. PLoS ONE.

[52] Dehne N., Brune B. (2009). HIF-1 in the inflammatory microenvironment. Exp. Cell Res..

[53] Li G.Y., Fan B., Wu Y.Z., Wang X.R., Wang Y.H., Wu J.X. (2005). Changes of expression of HIF-1α in the human retinal pigment epithelium induced by hypoxia. Zhonghua Yan Ke Za Zhi.

[54] Arjamaa O., Aaltonen V., Piippo N., Csont T., Petrovski G., Kaarniranta K., Kauppinen A. (2017). Hypoxia and inflammation in the release of VEGF and interleukins from human retinal pigment epithelial cells. Graefes Arch. Clin. Exp. Ophthalmol..

[55] Kalhori V., Kemppainen K., Asghar M.Y., Bergelin N., Jaakkola P., Tornquist K. (2013). Sphingosine-1-phosphate as a regulator of hypoxia-induced factor-1α in thyroid follicular carcinoma cells. PLoS ONE.

[56] Strieter R.M., Kunkel S.L., Elner V.M., Martonyi C.L., Koch A.E., Polverini P.J., Elner S.G. (1992). Interleukin-8. A corneal factor that induces neovascularization. Am. J. Pathol..

[57] Wilkerson B.A., Grass G.D., Wing S.B., Argraves W.S., Argraves K.M. (2012). Sphingosine 1-phosphate (S1P) carrier-dependent regulation of endothelial barrier: High density lipoprotein (HDL)-S1P prolongs endothelial barrier enhancement as compared with albumin-S1P via effects on levels, trafficking, and signaling of S1P1. J. Biol. Chem..

[58] Blaho V.A., Galvani S., Engelbrecht E., Liu C., Swendeman S.L., Kono M., Proia R.L., Steinman L., Han M.H., Hla T. (2015). HDL-bound sphingosine-1-phosphate restrains lymphopoiesis and neuroinflammation. Nature.

[59] Christensen P.M., Liu C.H., Swendeman S.L., Obinata H., Qvortrup K., Nielsen L.B., Hla T., di Lorenzo A., Christoffersen C. (2016). Impaired endothelial barrier function in apolipoprotein m-deficient mice is dependent on sphingosine-1-phosphate receptor 1. FASEB J..

[60] Lambert V., Lecomte J., Hansen S., Blacher S., Gonzalez M.L., Struman I., Sounni N.E., Rozet E., de Tullio P., Foidart J.M. (2013). Laser-induced choroidal neovascularization model to study age-related macular degeneration in mice. Nat. Protoc..

